# Management of acute respiratory diseases in the pediatric population: the role of oral corticosteroids

**DOI:** 10.1186/s13052-017-0348-x

**Published:** 2017-03-23

**Authors:** Renato Cutrera, Eugenio Baraldi, Luciana Indinnimeo, Michele Miraglia Del Giudice, Giorgio Piacentini, Francesco Scaglione, Nicola Ullmann, Laura Moschino, Francesca Galdo, Marzia Duse

**Affiliations:** 1 Pediatric Pulmonology and Sleep & Long Term Ventilation Unit, Academic Department Pediatric Hospital “Bambino Gesù”, Piazza S. Onofrio 4, 00165 Rome, Italy; 2 Women’s and Children’s Health Department, University of Padua, Via Giustiniani 3, 35128 Padova, Italy; 3 Department of Maternal and Child Care and Urology, Gender Medicine Polyclinic, University of Rome “Sapienza”, Piazzale Aldo Moro 5, 00185 Rome, Italy; 4 Department of Woman, Child and General and Specialized Surgery, Second University of Naples, Via Luigi De Crecchio 4, 80138 Naples, Italy; 5 Department of Surgery, Dentistry, Paediatrics and Gynecology, University of Verona, Policlinico G.B. Rossi, Piazzale L.A. Scuro 10, 37134 Verona, Italy; 6 Department of Oncology and Onco-Hematology, University of Milan, Via Vanvitelli 32, 20129 Milan, Italy

**Keywords:** Acute respiratory diseases, Asthma, Bronchiolitis, Croup, Respiratory failure, Wheezing

## Abstract

Respiratory diseases account for about 25% of all pediatric consultations, and 10% of these are for asthma. The other main pediatric respiratory diseases, in terms of incidence, are bronchiolitis, acute bronchitis and respiratory infections. Oral corticosteroids, in particular prednisolone, are often used to treat acute respiratory diseases given their anti-inflammatory effects. However, the efficacy of treatment with oral corticosteroids differs among the various types of pediatric respiratory diseases. Notably, also the adverse effects of corticosteroid treatment can differ depending on dosage, duration of treatment and type of corticosteroid administered — a case in point being growth retardation in long-course treatment. A large body of data has accumulated on this topic. In this article, we have reviewed the data and guidelines related to the role of oral corticosteroids in the treatment and management of pediatric bronchiolitis, wheezing, asthma and croup in the attempt to provide guidance for physicians. Also included is a section on the management of acute respiratory failure in children.

## Background

### The burden of acute respiratory diseases in children in Italy

Acute and chronic respiratory diseases represent a global public health problem because of their increasing prevalence and severity worldwide [1]. This can be attributed to several factors: (i) the significant increase in the prevalence of early allergen sensitization in childhood; (ii) the frequent recurrence of viral infections typically associated with children; and (iii) the increased survival of extremely preterm and fragile children born with bronchopulmonary dysplasia. All these factors contribute to the increased risk of acute manifestations becoming chronic. Also lung function persistently deteriorates thereby leading to the development of chronic respiratory diseases in adulthood.

Epidemiological data on the prevalence of respiratory diseases are scarce. Reliable data for Italy come from the SIDRIA (Italian Studies on Respiratory Disorders in Childhood and the Environment) study. Conducted on over 20,000 children 6–7 years old and on 16,000 adolescents in two phases (1994 and 2002), the SIDRIA study showed a clear increase in the prevalence of asthma in both groups [2, 3]. A much more recent study conducted in Rome confirmed these prevalence data in a population of preschool children (3–5 years old): 15% of children experienced at least one episode of wheezing, and 11% had a doctor’s diagnosis of asthma [4]. The frequency of allergic sensitization in this age group was already as high as 12%, while, currently, the prevalence of allergic sensitization abundantly exceeds 30% of the 10–14-year-old population, with peaks exceeding 40%. All these data confirm, in Italy, the general worldwide trend of the increasing frequency of allergic sensitization, which is a major factor in the occurrence of respiratory diseases, especially asthma.

If data on the prevalence of allergic sensitization in Italy are scarce, there are no recent data at all about the impact of acute respiratory diseases on hospital or emergency department admissions. To access numerical data, we must turn to the international literature. In a retrospective chart review of discharges from the Medical University of South Carolina from 1956 to 1997, the primary discharge diagnosis of asthma increased 24-fold in black children versus 5-fold in white children [5]. This trend confirmed a previous study [6] and was in line with a subsequent report that similar increases were taking place in all industrialized countries [7]. The causes of this increase are several, but the major cause is undoubtedly allergic sensitization acting synergistically with viral infections in the development of acute and chronic respiratory diseases.

### Risk factors

Kusel et al. [8] studied a cohort of 263 at-risk children (at least one parent was atopic) and found that atopic sensitization within the first 2 years of life was the most important risk factor for developing asthma at 5 years. Suffering from more than two episodes of either Rhinovirus or Respiratory Syncytial Virus infection, or from a single infection of the lower respiratory tract was accompanied by a 7- and a 5-fold greater risk, respectively, of developing asthma versus their not atopic peers or peers who developed allergic sensitization later in life. The COAST study [9] confirmed these observations. In fact, in 298 at-risk newborns, both univariate and multivariate analyses showed that the prevalence of asthma at age 14 years was 6-fold higher in subjects who were allergic at one year of age. Allergic sensitization became a progressively less significant risk factor with increasing age and was irrelevant in children > 5 years.

Viral infections have been consistently associated with wheezing (‘viral wheezing’) [10, 11]. More recently, bacterial infections were found to be significantly associated with acute wheeze in young children (4 weeks–3 years) in a manner similar to viral infection but independently [12]. In summary, acute severe lower respiratory infections caused by viruses or bacteria in the first years of life are important contributors to current asthma and persistent wheeze in older children sensitized in the first 2 years of life.

Emerging data support the hypothesis that priming of the immune system to allergens may occur even earlier, namely, during antenatal life. It is well established that predisposition to produce IgE in response to environmental stimuli depends on both genetic and environmental factors. These interactions probably begin in utero and, through regulatory mechanisms conditioned by the environment (‘epigenetic mechanisms’), they may influence the onset, expression and phenotypes of diseases at later ages or even in adulthood [13]. The hygiene hypothesis [14] can provide an interpretive key in this context. In fact, our environment and the air we breathe can be rich or poor in bacterial endotoxins. Our mucous membranes (nose, lung and intestine) colonized by millions of germs can be rich or poor in several pathogenic or non-pathogenic species. Mucosal colonization is the most powerful immunological factor that can affect immune system function and induce appropriate immune responses as the need arises (normal model), or, mainly, IgE-mediated (allergy) or cytotoxic (chronic/inflammatory autoimmune diseases) inflammatory responses [15–17]. Thus, children born and living in rural areas, close to stables and animals, suffer less from asthma and allergies than their urban counterparts, because in their early years (even in utero) they are exposed to non-pathogenic stimuli, i.e., environmental endotoxins [18]. Furthermore, the hygiene hypothesis is in line with the finding that the offspring of vaccinated mothers have a ‘better’ gut colonization than the offspring of non-vaccinated mothers thanks to the broader antibody repertoire (in terms of titer and repertoire) passively transferred to them in the last weeks of pregnancy [19]. Finally, it is clear that breastfeeding may not only play an anti-inflammatory role, but may also drive microbiota colonization and mucosal immune maturation [20]. These processes are all closely integrated and lead to different ‘imprinting’ of the immune system and thus condition the predisposition to develop diseases, allergic or not.

With the advent of epigenetics, it became clear that our genetic heritage is in fact plastic and any alterations induced by DNA methylation/demethylation changes can become permanent and be transmitted to offspring [21]. Attempts to translate these acquisitions in preventive measures have been disappointing. Containment of indoor and outdoor pollution improves the clinical course of lung disease, but impacts little on their frequency [22]. The high concentration of endotoxins in the environment is highly protective, but if associated with a high rate of pollution, its protective effect can be reverted [22]. Therefore, preventive measures must not be limited to primary prevention (namely, healthy and not polluted environments, breastfeeding, a diet rich in vegetables/fruit, and an intensive and active immunization policy). In fact, inflammation should be treated to halt its progression or chronicity. Only strategies based on a broad range of treatment protocols, including different classes of anti-inflammatory drugs or immunomodulatory therapies, may result in disease modification [23].

Herein, we discuss critical issues related to asthma and to the treatment of the acute respiratory diseases. We do not address forms of respiratory diseases, such as pneumonia, that have a different pathogenic basis and therefore require a very specific approach.

## Pharmacology of corticosteroids

### Anti-inflammatory action

Given their anti-inflammatory effects, glucocorticoids are among the most frequently prescribed agents in clinical practice with indications that, over time, have been extended to various diseases. Glucocorticoids have a very broad spectrum of anti-inflammatory activity that involves both the humoral and cellular systems (Table 1). They prevent or suppress inflammation in response to many stimuli including immunological, radiant, mechanical, chemical and infectious stimuli [24].

**Table 1:** Effects exerted by glucocorticoids on cells, and factors involved in the inflammatory response

Cells	Factors involved in the inflammatory response	Comments
Macrophages and monocytes	Cascade of arachidonic acid (prostaglandins and leukotrienes)	Mediated by inhibition of PLA2 and reduced COX-2 expression.
	Inflammatory cytokines (e.g., IL-1,2,4,5,6,11,13) and TNF-α	Reduced production and release.
		Cytokines exert multiple effects on inflammation, e.g., T-cell activation and stimulation of fibroblast proliferation.
Endothelial cells	ELAM-1 and ICAM-1	ELAM-1 and ICAM-1 are important for extravasation of leukocytes into tissues.
Basophils	Histamine and LTC_4_	IgE-dependent release inhibited by glucocorticoids.
Fibroblasts	Arachidonic acid	See “Macrophages and monocytes”. Glucocorticoids also reduce the proliferation of fibroblasts.
Lymphocytes	Cytokines (IL-1, IL-2, IL-3, IL-6, TNF-α, GM-CSF, interferon-γ)	See “Macrophages and monocytes”.

*COX-2* cyclooxygenase-2, *ELAM-1* endothelial-leukocyte adhesion molecule-1, *ICAM-1* intercellular adhesion molecule-1, *IL* interleukin, *LTC*_*4*_ leukotriene C4, *PLA2* phospholipase A2, *TNF-α*, tumor necrosis factor-α

### Mechanism of action

Corticosteroids bind to specific intracellular receptors in target tissues to regulate the expression of corticosteroid-responsive genes, thereby changing the levels of proteins synthesized by target tissues. Given the time required to modulate gene expression and protein synthesis, most of the genomically mediated effects of corticosteroids are not immediate. This is clinically important because the beneficial effects of corticosteroid therapy are manifested with a certain delay [25]. On the contrary, the effects of corticosteroids exerted via a non-genomic mechanism, e.g., via steroid-selective membrane receptors, are manifested immediately [25–28].

### Pharmacokinetics

All corticosteroids are well absorbed and effective when delivered by the oral route. Some water-soluble esters of hydrocortisone and its synthetic congeners can be administered intravenously to rapidly obtain high concentrations of the drug in body fluids. Glucocorticoids can also be absorbed systemically from sites of local administration (i.e., the respiratory tract). After absorption, ≥90% of plasma cortisol is reversibly bound to proteins. Only the fraction of free corticosteroid is active and can enter cells. Two plasma proteins represent almost the entire binding capacity of steroids: corticosteroid-binding globulin (also called ‘transcortin’) and albumin. All biologically active corticosteroids are extensively metabolized in the liver. Synthetic steroids with an 11-keto group, such as cortisone and prednisone, must be enzymatically reduced to the corresponding 11β-hydroxy derivative to be biologically active. In conditions in which this enzymatic activity is deranged, it is advisable to use steroids that do not require enzymatic activation (i.e., hydrocortisone or prednisolone). Such conditions include severe liver failure, defects of hexose-6-phosphate dehydrogenase, and the rare condition of cortisone reductase deficiency [27]. According to the GINA (Global Initiative for Asthma) Guidelines, oral glucocorticoid administration is as effective as intravenous administration [29]. However, different oral formulations may have different pharmacokinetic profiles; for instance, compared to the tablet formulation, liquid prednisolone results in significantly higher plasma concentrations immediately after administration [30]. Moreover, prednisolone solution produces a 20% higher peak plasma level of prednisolone approximately 15 min earlier than tablets [31]. The liquid preparation is therefore a suitable alternative for children and patients unable to swallow tablets [30].

### Glucocorticoids in medical practice

Attempts have been made to increase the anti-inflammatory effect of glucocorticoids by modifying their molecular structure. In many cases, these synthetic agents exert an enhanced anti-inflammatory effect and their action is more prolonged. The most widely used synthetic glucocorticoids in medical practice are listed in Table 2 [24, 27, 32]. Oral corticosteroids are classified based on their anti-inflammatory potency, which corresponds to the duration of action, and on the potency of the suppressive hypothalamic-pituitary-adrenal (HPA) axis. Equivalent doses of each corticosteroid administered in clinical practice are calculated taking into account the different anti-inflammatory potencies, and exert the same anti-inflammatory effects. Notably, both the genomic- and non-genomic effects exerted by the various types of corticosteroids differ widely in terms of their relative potencies. For example, betamethasone has a very low non-genomic potency versus prednisolone but a higher genomic potency, which suggests that prednisolone has a faster onset of action [26]. These characteristics can serve to define the clinical efficacy/tolerability profile of each molecule.

**Table 2:** Classification and comparison of the major systemically used glucocorticoids

Glucocorticoid	Equivalent dose (mg)	Anti-inflammatory potency*	Salt retention*	Suppressive HPA potency*	Biological half-life (h)	
Hydrocortisone	20	1	1	1	8–12	Short
Cortisone	25	0.8	0.8	1	8–12	Short
Prednisolone	5	4	0.8	1	12–36	Intermediate
Prednisone	5	4	0.8	1	12–36	Intermediate
Methylprednisolone	4	4–5	0.2–0.5	5	12–36	Intermediate
Triamcinolone	4	5–10	0	5	12–36	Intermediate
Dexamethasone	0.75	25	0	50	36–72	Long
Betamethasone	0.75	25	0	50	36–72	Long

*Hydrocortisone as reference drug set equal to 1

*HPA* hypothalamic-pituitary-adrenal axis

### Tolerability

The most frequent adverse events of corticosteroids are mainly associated to the suppression of the HPA axis. Consequently, their use should be carefully evaluated in terms of the benefit/risk ratio [24]. In children, corticosteroids have been associated to various adverse effects, mainly in terms of dosage, type of glucocorticoid and treatment duration [33]. The most frequently observed adverse reactions associated with short-course oral corticosteroids are vomiting, behavioral changes, infections and disturbed sleep, while growth retardation is associated with prolonged treatment [34]. Repeated short courses of oral prednisone were not associated with any lasting perturbation in bone metabolism, bone mineralization, or adrenal function [35].

### Withdrawal symptoms

Treatment for 3-6 days with corticosteroids that have a short or intermediate biological half-life (e.g. prednisolone or prednisone) is not associated with withdrawal complications. Withdrawal syndrome is characterized by the appearance of non-specific symptoms when prolonged corticosteroid treatment is stopped abruptly, and in very rare cases, also when treatment is discontinued gradually. The symptoms and the most common signs of corticosteroid withdrawal are anorexia, nausea, vomiting, severe asthenia, arthromyalgia, headache, weight loss, depression and lethargy [24, 27].

### Adrenocortical insufficiency

Chronic treatment with systemic corticosteroids (SCs) can induce suppression of the HPA axis. As a consequence, the adrenal gland may not be able to produce adequate amounts of cortisol upon discontinuation of treatment. This secondary adrenal insufficiency is often overlooked in non-symptomatic cases. It is important to identify asymptomatic patients because exogenous stress (trauma, illness or surgery) may precipitate a severe acute hypoadrenal crisis. Corticosteroid administration in the initial stage of stress can prevent this crisis. The pathogenesis of adrenal insufficiency secondary to corticosteroid therapy is complex. Moreover, it varies from patient to patient and has not yet been fully clarified, especially in terms of predisposing factors. The symptoms and signs of adrenal insufficiency are non-specific (Table 3) and its diagnosis can be confirmed with the adrenocorticotropin (ACTH) stimulation test. However, the ACTH test cannot identify the rare cases of adrenal insufficiency due to central suppression of the HPA axis. In such cases, a more specific test (the corticotropin-releasing hormone test) is necessary [24].

**Table 3:** Signs and symptoms suggestive of adrenal insufficiency

Cardiovascular instability	
Discrepancy between disease severity and clinical status of the patient presenting nausea	
Orthostatic hypotension	
Dehydration	
Lower abdominal pain or weight loss	
Fever of unknown origin	
Apathy, depression not related to psychiatric illness	
Altered pigmentation, loss of axillary and pubic hair	
Hypothyroidism and hypogonadism	
Hypoglycemia, hyponatremia and hyperkalemia	
Neutropenia and eosinophilia	

### Factors favoring suppression of the HPA axis

Suppression of the HPA axis depends on several factors; an understanding of these factors can help to reduce the risk of this condition.

#### The glucocorticoid used

The compounds used to treat patients differ in biological characteristics and indications. The intrinsic potency and biological half-life of each compound correlate with the ability to induce suppression of the HPA axis. The longer the biological half-life, the more prolonged the ACTH suppression after a single dose (Table 2). Multiple daily doses reduce the time required for the HPA axis to recover full efficiency, thereby resulting in an increased risk of suppression of the HPA axis. In routine practice, preference should be given to synthetic analogs (prednisone and prednisolone) that have a good balance between potency and HPA axis-inhibiting effect. Greater potency and a longer biological half-life (e.g., betamethasone and dexamethasone) may result in a stronger inhibition of the HPA axis even after the administration of a single dose. On the other hand, intermediate compounds such as prednisolone exert very little and transient suppression of the axis with no negative feedback on either the pituitary gland or hypothalamus, as shown by the absence of altered ACTH production [32].

As shown in Table 2, the betamethasone:prednisolone equivalent dose ratio is 25:4; thus, 5 mg of prednisolone are required to obtain the same efficacy as 0.75 mg of betamethasone. However, the inhibitory effect of the betamethasone:prednisolone ratio on the HPA axis is 50:1; thus 250 mg of prednisolone are required to obtain an inhibitory potency of 1 mg of betamethasone on the HPA axis! [36].

#### The glucocorticoid administration schedule

Endogenous cortisol production peaks early in the morning and gradually declines until evening. Consequently, evening administration of a corticosteroid would disrupt the normal ACTH circadian cycle. Therefore, corticosteroids should be preferably administered in a single dose in the morning. When, for reasons of corticosteroid type or for clinical reasons, twice daily dosing is preferred, two-thirds of the dose may be given in the morning and one-third in the afternoon. This administration regime mimics the normal circadian rhythm of adrenal secretion and decreases the risk of inhibition of the HPA axis.

#### Route of administration

The risk of corticosteroid-induced suppression of HPA axis function is much lower with topical administration than with oral administration. Inhaled corticosteroids are generally considered ‘low risk’ and are the first choice in many diseases (e.g., asthma).

#### Duration of corticosteroid treatment and cumulative dose

Although epidemiological data are scarce and inconclusive, the duration of corticosteroid treatment and the cumulative dose are two important risk factors that predict HPA axis function. From a practical viewpoint, treatment with prednisolone or prednisone at a dose of 5 mg/day for 10–15 days entails a very low or no risk of HPA axis inhibition. Obviously, high power and long biological half-life corticosteroids (betamethasone and dexamethasone) inhibit the HPA axis in less time.

### How to reduce the risk of HPA suppression

Although it is difficult to predict HPA suppression in individual patients, the following strategies may reduce this risk.

1. Use SCs for well-documented indications.
2. Prefer a medium-acting analog (prednisolone or prednisone).
3. Use the lowest effective dose for the shortest duration possible and administer the drug in a single dose in the morning or, when possible, on alternate days.

There are no reliable data about the correct way of discontinuing treatment but only expert opinions and therapeutic practice established by tradition. Whenever possible, the corticosteroid dose should be progressively reduced, and the patient should be monitored for disease exacerbation, secondary adrenocortical insufficiency, or withdrawal syndrome.

### Recovery of the HPA axis

Functional recovery of the HPA axis, especially when administering intermediate half-life corticosteroids, can be obtained by lengthening inter-dose intervals. Where possible, the compounds with an intermediate potency and biological half-life (prednisolone and prednisone) should preferably be administered on alternate days. However, this strategy has no advantage in case of high potency compounds and compounds with a long biological half-life, because they prolong inhibition of the HPA axis. The time of ’recovery’ of the HPA axis after discontinuing treatment may vary from individual to individual, and it may take from weeks to months. If the dosage of steroids is gradually reduced, true adrenal insufficiency is rare, at least in the absence of stress. There is no demonstration that ACTH treatment can accelerate functional recovery of the HPA axis. Its use for this purpose is therefore not justified.

### Summary

**Table:** 

ᅟ	ᅟ
• Glucocorticoids prevent or suppress inflammation in response to immunological, radiant, mechanical, chemical and infectious stimuli. • Oral glucocorticoids are well absorbed and effective. • Glucocorticoids have been associated with adverse effects, mainly in relation to dosage, type of glucocorticoid and treatment duration. • Prolonged glucocorticoid treatment can be associated with suppression of HPA axis function and growth retardation. • Administration of glucocorticoids with a short or intermediate biological half-life (e.g., prednisolone or prednisone) for 3–6 days is not associated with withdrawal symptoms.	

## Bronchiolitis

### Epidemiology, age frequency and seasonality

Viral bronchiolitis is the most frequent cause of lower respiratory tract infection and the leading cause of hospitalization in infants in the first year of life (approximately 1% of children in Europe and the United States) [37–40]. The highest age-specific rate of respiratory syncytial virus (RSV) hospitalization seems to occur in infants between 30 days and 60 days of age, with contrasting rates reported for preterm-born children [38]. The disease is associated with a high mortality rate in developing countries, but even in industrialized countries it is the main cause of death due to viral infection during the first year of life [37]. The most common pathogen is RSV, which accounts for 60–80% of all cases [38, 39], with a peak incidence from November to April. Also human rhinovirus (HRV), parainfluenza virus and metapneumovirus are frequently involved in bronchiolitis, with a variable seasonality [37–39].

### Main risk factors

Severe forms of bronchiolitis requiring hospitalization may be more frequent in children below 3 months of age (which corresponds to the natural nadir of postnatal maternal immunoglobulins) and in preterm-born children (particularly those born <32 gestational weeks), whose immune system is still immature [37, 38, 40]. Infants with such chronic lung diseases as bronchopulmonary dysplasia and cystic fibrosis, and those with hemodynamic congenital heart diseases are also at an increased risk of severe bronchiolitis [37, 38, 40]. Other high risk populations are children with congenital neuromuscular diseases and with malformation syndromes or sequences (e.g., Pierre-Robin, CHARGE and Jeune syndrome), patients with Down syndrome, because of the underlying heart disease and their state of relative T lymphocyte immunodeficiency, and patients with primary or secondary immunodeficiency (such as Di George or Wiskott-Aldrich syndrome, neonatal HIV and transplant recipients) [37]. Finally, male gender, crowding, lack of breast feeding, low socio-economic status, exposure to indoor smoke pollution and daycare attendance are also possible risk factors [38, 40].

### Clinical presentation and severity

Various definitions of bronchiolitis have been proposed, but the term is generally applied to a first episode of wheezing and crackles in infants below 12 months of age [37, 38, 40]. Children with acute bronchiolitis may present with a wide range of clinical symptoms, therefore a detailed physical examination and clinical history taking are required [37]. Typically, an infant with bronchiolitis presents during the epidemic season after 2-4 days of low grade fever, nasal congestion and rhinorrhea, possibly after exposure to persons with upper respiratory tract viral infection [37–39]. Symptoms of lower respiratory tract illness may include:

- Cough.
- Tachypnea or apnea (especially in preterm infants below the age of 3 months).
- Increased respiratory effort (grunting, nasal flaring, and intercostal, subcostal or supraclavicular retractions).
- Inspiratory crackles and expiratory wheezing at auscultation.
- Low oxygen saturation (SatO2).
- Dehydration due to feeding difficulties.

In addition, disease severity must be assessed to identify children who require hospital admission (Table 4).

**Table 4:** Factors affecting the decision to admit children to the hospital

	Possible discharge	Brief observation	Hospitalization
Respiratory effort	None or mild chest wall retraction	Tracheal tug, nasal flare, Moderate chest wall retraction	Moderate-to-severe respiratory distress, apnea
Oxygen saturation	No supplemental oxygen requirement, saturations > 95%	Saturations 90–95%	Saturations persistently < 90–92%, O2 requirement
Feeding	Normal to slightly decreased	50–75% of normal feeds	<50% of feeds, unable to feed, dehydration
Gestational age	Gestational age >37 weeks, birth age >12 weeks		Gestational age <37 weeks, birth age <6–12 weeks
Responsivity and alertness	Reactive, vigilant		Less or not responsive
Social factors	Good parent compliance, hospital easily accessible		Non-collaborative parents, Distant from hospital
Preexisting risk factors	No risk factors	BPD, cystic fibrosis, cyanogenic congenital heart disease, immunodeficiency, neuromuscular disease	BPD, cystic fibrosis, cyanogenic congenital heart disease, immunodeficiency, neuromuscular disease

*BPD* bronchopulmonary dysplasia

### Management and treatment

**Fig. 1:**
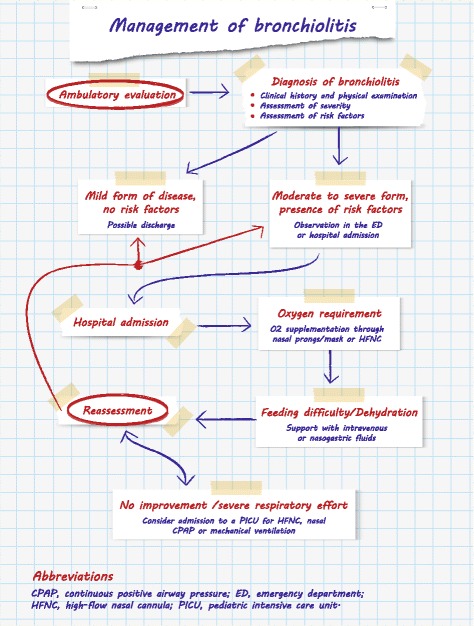
Management of bronchiolitis

The management of bronchiolitis largely depends on the severity of the condition (Fig. 1). However, the best therapeutic approach to the different stages of bronchiolitis is controversial. Despite the wide array of pharmacological treatment options, oxygen supplementation and supportive therapy to control hydration remain the mainstay of treatment [37, 38, 40]. Supplemental oxygen should be administered if O_2_ saturation levels are persistently below 90–92% at ambient air [37, 38, 40], with SatO2 measured by pulse oximetry using pediatric probes and after suctioning of the nares [37, 41]. Oxygen may be administered by means of nasal prongs or a face mask, but in case of increased respiratory effort, high-flow oxygen therapy with humidified and heated oxygen (high-flow nasal cannula, HFNC) should be considered, even in a pediatric ward setting [37, 41]. Intravenous or nasogastric fluids may be used for children with bronchiolitis who cannot maintain oral hydration; the two routes of administration are similarly effective [37, 38, 40].

Regarding pharmacological therapy, according to the most recent guidelines and documents on bronchiolitis management, there is no evidence supporting the use nebulized adrenaline, salbutamol, ipratropium bromide, antibiotics, antivirals, or systemic or inhaled corticosteroids in routine practice [37, 38, 40]. However, hypertonic saline may be administered in infants to decrease airway edema and improve mucociliary clearance [38]. Despite evidence that corticosteroids are beneficial in other respiratory diseases, large reviews have shown that, in infants with bronchiolitis, neither systemic nor inhaled steroids decrease the incidence or duration of hospitalization, neither do they improve the short- and long-term prognosis [37, 38, 42]. Several trials have compared the effect of oral corticosteroids administered for different durations and at different dosages versus placebo in bronchiolitis management [37, 38, 40, 42]. The association of systemic (oral) corticosteroids with epinephrine or bronchodilator therapy has also been evaluated, but according to the National Institute for Health and Care Excellence [40], corticosteroids, whether administered alone or combined with epinephrine or bronchodilator therapy, do not produce a significant benefit [40]. On the other hand, in a randomized trial, oral dexamethasone associated to salbutamol shortened by a mean of 31% the duration to readiness for discharge during bronchiolitis episodes in patients with eczema or with a family history of asthma in a first-degree relative [43]. Further studies are needed to confirm the latter finding which was obtained in a selected population of infants at high risk of asthma.

### Environmental and pharmacological prophylaxis

Environmental prophylaxis (with frequent hand washing, and decontamination of garments, toys and medical instruments) is necessary to decrease the burden of viral bronchiolitis, especially in the hospital setting. Pharmacological prophylaxis with the humanized monoclonal antibody palivizumab for high risk populations reduces the possibility of developing a severe form of the disease [37–40, 44]. Given the paucity of therapeutic alternatives, prevention strategies for RSV infection are crucial, and on the horizon there are new vaccines and monoclonal antibodies that have an extended half-life of 70–100 days, and can thus be administered in a single injection for the whole season. Maternal RSV vaccination is particularly relevant because hospitalizations peak at 2–3 months of age, when children are unlikely to benefit from active immunization. Given the role of RSV in the pathogenesis of recurrent wheeze and asthma, the importance of monoclonal antibodies and vaccines could extend beyond the prevention of hospital admission of infants to the long-term respiratory health and primary prevention of asthma [39, 44].

### Summary

**Table:** 

ᅟ	ᅟ
• Bronchiolitis is the most frequent cause of lower respiratory tract infection in the first year of life, with 60–80% of cases caused by RSV, and with the highest rate of hospitalization occurring in infants < 2–3 months of age. • Infants < 3 months old, preterm infants, infants with chronic lung diseases (bronchopulmonary dysplasia or cystic fibrosis), congenital heart or neuromuscular diseases, and infants with immunodeficiency have an enhanced risk of hospitalization. • Cough, rhinorrhea, low grade fever, tachypnea or apnea, and symptoms of lower tract respiratory infection (grunting, chest retractions, crackles and wheezing) may be present. • Oxygen supplementation and supportive therapy to control hydration remain the mainstay of treatment. • There is no evidence supporting the use of salbutamol, nebulized adrenaline or inhaled or oral corticosteroids in routine practice. • RSV bronchiolitis, especially when severe, increases the risk of developing recurrent wheezing and asthma later in life.	

## Wheezing in the preschool child

Given its high prevalence, wheezing in preschool age is a major issue for pediatricians in terms of diagnosis and management. It has been suggested that one-third of preschoolers may present wheezing before the age of 5 years [45] and this condition may be either episodic or persistent, with different levels of severity. Although preschool wheezing usually resolves by school age, and only a minority of preschool children with wheezing is expected to develop persistent asthma, most school-age asthmatic children suffer from wheezing before the age of six years [3].

### Phenotypic classifications

Because of the different clinical and pathophysiological features of children affected by wheezing, preschool wheezing is usually approached from a phenotypic perspective. In a symptom-based classification of the causal triggers of wheezing episodes, ‘episodic viral wheezing’ without symptoms between exacerbations was differentiated from ‘multiple-trigger wheeze’ with symptoms between episodes [46]. In a time-trend-based classification, childhood wheezing was classified ‘transient wheeze’, ‘persistent wheeze’ or ‘late onset wheeze’ [47]. The advantage of a phenotypic classification is that patients are classified according to their basic characteristics in an early fashion and can thus undergo timely treatment. However, given the evidence of phenotype instability and overlapping, it has recently been proposed that wheezing in preschoolers be classified according to frequency and severity of symptoms.

### Asthma in preschoolers: diagnosis

The 2016 GINA guidelines [29] acknowledged that asthma may be clinically evident in children below the age of 5 years. Given that a definite diagnosis of asthma in preschool children may be complicated by such confounding factors as recurrent respiratory tract infections, and given the paucity of tests available for this age group, the GINA expert panel devised a tentative risk characterization for a diagnosis of asthma in children focused on symptom severity and frequency, personal and family characteristics suggestive of atopy, and response to trial treatment with inhaled corticosteroids.

### Treatment of wheezing in preschoolers

According to the GINA guidelines [29] while short-acting beta2 agonists are recommended for all asthma patients when needed, therapeutic strategies in preschool children with recurrent wheezing should be frequently reviewed and be tailored to the characteristics of each child because he/she may be affected by early asthma as opposed to transient viral wheezing. Regular therapy with inhaled corticosteroids is the first option in preschool children presenting with recurrent wheezing if episodes are frequent and/or severe, or if interval symptoms are reported [29]. Therefore, while inhaled corticosteroids were previously administered only in young children who presented with early signs of asthma, the most recent position documents recommend inhaled corticosteroids also in viral wheezers, either with low-dose regular treatment or with a high-dose intermittent strategy [29, 48]. The GINA document [29] confirms the use of SCs in the treatment of severe exacerbations in children aged 5 years or younger.

Oral corticosteroids are indicated in closely monitored children with severe exacerbations at a dose equivalent to prednisolone 1–2 mg/kg/day, with a maximum of 20 mg/day in children below the age of 2 years, and 30 mg/day in children aged 2–5 years. Treatment is recommended for 3–5 days and can be abruptly halted. The bitter taste of most oral corticosteroid preparations may hamper compliance in preschool children. In a group of 78 children, mean age 24 months, with acute asthma exacerbation, oral liquid prednisolone was better tolerated than crushed tablets administered with lemonade or custard. It resulted in less vomiting and had a similar level of efficacy [49]. Although early family/carer-initiated oral corticosteroids is widely practiced in some countries, the GINA document questions this practice because of scarce evidence of its effectiveness in the home management of wheezing episodes in preschoolers. Moreover, the GINA guidelines underline that self-treatment may be considered only if the physician is confident that carers are capable of using the medications appropriately, and if the child is closely monitored for side effects.

The use of SCs, given for decades to treat wheezing in young children, was based on an extrapolation from studies conducted in older children and adolescents affected by bronchial asthma who promptly and effectively responded to treatment with this class of drugs. The response to oral corticosteroids may differ depending on age and on the different phenotypes of wheezing and asthma in early childhood. In fact, in preschool wheezers, airway inflammation is mostly due to a neutrophilic response to viral triggers, while school-age children more frequently present with eosinophilic, highly steroid-responsive airway infiltration [50].

To better address the practical approach to clinical conditions, a number of studies evaluated the efficacy of treatment with oral steroids in acute wheezing exacerbations in preschool children. In a study performed in the early nineties in 74 emergency room children, aged 7–54 months, a single dose of intramuscular methylprednisolone in association with inhaled salbutamol was significantly more effective than placebo in reducing hospitalization [51]. In 2003, Csonka and coworkers [52] showed that oral prednisolone given for 3 days reduced disease severity, length of hospitalization, and symptom duration in children aged 6–35 months with virus-induced wheezing. In a subsequent study of 700 children aged 10 months to 5 years presenting to hospital care for acute virus-induced wheezing, oral corticosteroids were not better than placebo for any of the outcomes considered [53]. This study confirmed the authors’ previous observation obtained in a community-based study that evaluated parent-initiated oral prednisolone administration for the treatment of virus-induced wheezing in preschool children [53]. An editorial accompanying the latter paper stressed the need to reduce overuse of oral corticosteroids in preschool children with acute viral wheezing, but acknowledged that prednisolone plays a role in the treatment of preschool children with atopy who have acute exacerbations and in those with severe episodic wheezing who may be candidates for admission to an intensive care unit [54].

In 2014, Jartti and coworkers investigated the short- and long-term efficacy of prednisolone for the first acute rhinovirus-induced wheezing episode [55]. They concluded that prednisolone may have a positive effect in children with a high viral load, although it cannot be routinely recommended for all young children with the first acute rhinovirus-induced wheezing episode. Very recently, Castro-Rodriguez et al. conducted a meta-analysis of 11 studies devoted to the role of SCs in preschool children presenting with recurrent exacerbations of wheezing [56]. No difference was found between oral corticosteroids and placebo in terms of primary outcomes (hospital admission, need for an additional course of systemic steroids, unscheduled visits and length of hospital stay). However, some studies conducted in emergency departments suggested a lower risk of hospitalization for patients treated with oral corticosteroids, and a reduced need for additional courses of SCs in the inpatient setting [56]. In contrast, outpatient children treated with oral corticosteroids had a higher hospitalization rate, suggesting that caution be exercised in initiating this treatment in outpatient children for whom a risk of more severe exacerbation cannot be excluded. The authors conclude that oral corticosteroid treatment may be more effective in some subgroups of wheezing children, depending on the severity of exacerbation, timing of administration, genetic predisposition, underlying inflammatory response, and specific viral infection by rhinovirus. Moreover, they do not consider it appropriate to formulate a broad clinical recommendation for the treatment of preschool wheezers with oral corticosteroids and suggest that additional studies with larger, properly characterized populations be conducted to address this issue. This concept is echoed by an editorial commenting on the “Dilemma of systemic steroids in preschool children with recurrent wheezing exacerbations” [57].

**Fig. 2:**
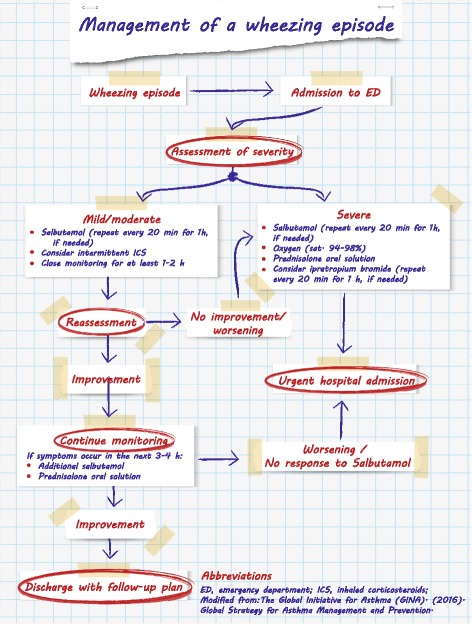
Management of a wheezing episode. Modified from: *The Global Initiative for Asthma (GINA). Global Strategy for Asthma Management and Prevention. 2016*

In conclusion, in preschool children, treatment with oral corticosteroids may be beneficial in those with frequent severe exacerbations, mostly those with an atopy background, and in those requiring emergency department or hospital admission and are closely monitored [29] (Fig. 2), while currently they are not considered to be indicated in preschool children with mild exacerbation of viral wheeze [48].

### Summary

**Table:** 

ᅟ	ᅟ
• Preschool wheezing is a heterogeneous condition that generally resolves by school age, but may represent the first manifestation of asthma in some children. • It is characterized by different clinical and pathophysiologic features. • Therapeutic strategies in preschool children with recurrent wheezing should be tailored to the frequency and severity of clinical manifestations. • The evidence supporting early family/carer-initiated oral corticosteroids in the home management of exacerbations is weak, and this treatment is not indicated for preschool children with mild exacerbation of viral wheeze. • According to the GINA guidelines, oral corticosteroids are indicated in children with severe wheezing exacerbations.	

## Asthma

Asthma is the most prevalent chronic disease of childhood and it results in considerable morbidity. It also interferes with normal activities and causes missed school days and much parental anxiety. It is characterized by airway inflammation and hyperresponsiveness to various stimuli [29, 58, 59]. How these features relate to each other and contribute to the clinical manifestations of asthma remains unclear. Important symptoms of asthma are wheezing, cough, difficulty breathing and chest tightness.

### Management of asthma in the emergency department

Asthma is one of the most common reasons for urgent care, emergency department visits and hospitalizations. Emergency department visits and hospitalizations are due to the severity of the asthma attack, but they are often due also to the lack of an asthma management plan or/and insufficient knowledge of how to deal with worsening on the part of the family. In the SIDRIA-2 study [3], the average annual rate of emergency department visits of children/adolescents with current asthma was 10% in the 12 months before study onset. Approximately 3% of children/adolescents with current asthma were admitted to hospital in the previous year, and over 30% were hospitalized at least once in their life.

Patients presenting to the emergency department with an asthma attack should be rapidly evaluated and triaged to assess the severity of symptoms and the need for important intervention [60, 61]. The patient’s history should be carefully collected, and a physical examination performed. Symptoms are poorly correlated with the severity of an asthma attack, therefore it is important to measure, if possible, the percentage of oxygen saturation (SpO2%) in ambient air and the peak expiratory flow or forced expiratory volume in 1 s (FEV1). The partial pressure of carbon dioxide (PaCO2) should be measured in case of a severe asthma attack. In the emergency department, an initial assessment should be followed by a second assessment after one hour of treatment to evaluate accurately the severity of the attack (Table 5).

**Table 5:** Levels of severity of an asthma attack in children

Clinical signs	Mild	Moderate	Severe	Life-threatening
Able to talk	Able to talk in sentences	Cannot complete sentences	Able to pronounce a few words	-
Respiratory rate^a^	Normal	Increased	Greatly increased	Bradypnea/gasping
Heart rate^b^	Normal	Increased	Greatly increased	Fall in heart rate
Complexion	Normal	Pallor	Pallor/cyanosis	Cyanosis
Level of consciousness	Normal	Restlessness	Severe restlessness	Obtundation, drowsiness
Wheezing	Mild expiratory	Expiratory	Expiratory/Inspiratory	Silent chest
Use of accessory muscles of respiration	Absent	Mild	Moderate	Paradoxical respiratory movement
SpO2	>95%	92–95%	<92%	<90%
PaCO2 (mmHg)	<38	38–42	>42	>42
Peak expiratory flow	>80%	60–80%	<60%	Not measurable

Not all clinical signs are necessary to classify a given level of severity

^a^Normal values: at <2 months of age ≤60/min; at 2–12 months ≤50/min; at 1–5 years ≤40/min; at 6–9 years ≤30/min

^b^Normal values: at 2–12 months of age ≤160/min; at 1–2 years ≤120/min; at 3–8 years, ≤110/min

### Pharmacological treatment of an asthma attack

#### Short-acting beta2-agonists

Short-acting beta2-agonists (SABAs) represent the rescue medication of choice and should be taken as needed to reverse bronchoconstriction and relieve symptoms. Salbutamol is the first-line choice. Inhaled salbutamol should be administered immediately on presentation. Pressurized metered-dose inhalers (pMDIs) with holding chambers are the optimal drug delivery device for patients with asthma exacerbations [62]. Two to four puffs of salbutamol 100 mcg administered by a pMDI via a spacer might be sufficient for a mild asthma attack, whereas up to 10 puffs may be needed for more severe attacks, repeated if necessary every 20-30 min during the first hour of treatment, and then every one to four hours as needed. Children and families must be trained to ensure successful delivery of the drug. Inhalers should be sprayed into the spacer in individual puffs and inhaled immediately by tidal breathing, for four-five breaths. If it is not possible to administer SABAs by a pMDI and spacer, most guidelines recommend the use of nebulizers at 2.5 mg/dose, up to a maximum dose of 5 mg, to be repeated every 20–30 min during the first hour of treatment. SABAs have the same effects on lung function, rate of hospitalization and oxygenation irrespective of whether they are delivered by a pMDI or by a nebulizer. However, bronchodilators delivered by a pMDI result in a shorter stay in the emergency department and less adverse effects (tachycardia, palpitation, tremor, and hypokalemia) than bronchodilators delivered by nebulizers. Special nebulizers must be used in case of continuous nebulization (0.5–5 mg/kg/h).

Endovenous administration of salbutamol is recommended in case of a life-threatening acute asthma attack not responsive to inhaled salbutamol. The recommended regime is 10 mcg/kg in 10 min, followed by continuous infusion (0.2 mcg/kg/min) up to a maximum dose of 2 mcg/kg/min. This should be administered in an intensive care unit with continuous electrocardiogram monitoring and daily electrolyte monitoring. Serum potassium levels are often low after multiple doses of salbutamol and should be replenished.

#### Ipratropium bromide

Ipratropium bromide is an anticholinergic agent used, in addition to inhaled SABAs, to treat children with a moderate-to-severe asthma attack who respond poorly to SABAs. Repeated doses (250 mcg/dose) of ipratropium bromide should be given. The early addition of this drug to salbutamol reduces the rate of hospitalization and improves both FEV1 and the clinical score in 60–120 min. Moreover, it decreases the rate of nausea and tremor. The addition of ipratropium bromide to salbutamol does not reduce the duration of hospitalization [63].

#### Epinephrine hydrochloride

Epinephrine hydrochloride remains the drug of choice in life-threatening situations involving anaphylaxis. Epinephrine is not recommended for the routine treatment of an asthma attack. It can be used if inhaled or intravenous bronchodilators are not available.

#### Corticosteroids

Systemic corticosteroids should always be given in case of an asthma attack, except in patients with a mild attack who rapidly respond to initial therapy with inhaled SABA. The early use of corticosteroids plays an important role in the emergency room. The use, versus the non-use, of corticosteroids is associated with a more rapid improvement in lung function, fewer hospital admissions, shorter hospitalization, a lower rate of relapse after discharge from the emergency department, and a reduction in the need for SABA.

Oral and parenteral corticosteroids are similarly effective. Nevertheless, the GINA 2016 guidelines recommend the oral route because it is quicker, less invasive and less expensive [29]. In the early treatment of acute asthma attacks, international guidelines [29, 58, 59] recommend oral prednisolone (1-2 mg/kg/die, up to a maximum dose of 40 mg/die). The guidelines of the British Thoracic Society underline that prednisolone is the most widely used steroid in patients with chronic asthma [59], and there is no evidence that other steroids provide an advantage [29, 58, 59]. In children, and whenever patients may have difficulties in swallowing, a liquid formulation is preferred to tablets [29, 64].

Intravenous methylprednisolone (1-2 mg/kg/6–8 h, up to a maximum dose of 40 mg) or hydrocortisone (5–10 mg/kg/6–8 h) should be reserved for severely affected children who are unable to retain oral medication. Larger doses do not appear to have a therapeutic advantage in most children. Treatment with intermediate biological half-life oral corticosteroids (e.g., prednisolone) for up to three-five days is recommended, but treatment should be continued until the child recovers.

High-dose inhaled corticosteroids are associated with fewer emergency department visits and hospitalizations compared with placebo. These drugs are safe but expensive and their dosage is difficult to establish [65]. Therefore, there is no evidence supporting the use of inhaled corticosteroids as a substitute for oral corticosteroids in the treatment of an asthma attack [66].

#### Other therapies

Oral or parenteral leukotriene receptor antagonists are not recommended for the treatment of an asthma attack. They affect neither function nor the overall hospitalization rate. Methylxanthines can only be used, in addition to SABAs, corticosteroids and oxygen therapy, for the treatment of a severe asthma attack. Magnesium sulfate may be effective in children with a more severe asthma attack, however only a few studies have included children [67]. Heliox is a mixture of helium and oxygen, usually 70% and 30%, respectively. The role of heliox in the management of an acute pediatric asthma attack is unclear.

### Management of an asthma attack in children aged > 5 years

**Fig. 3:**
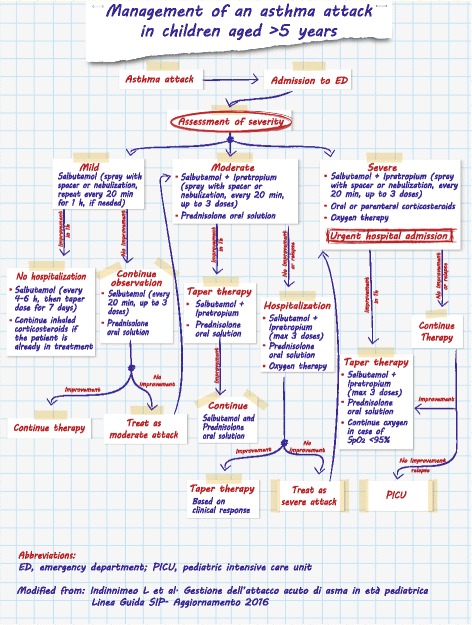
Management of an asthma attack in children aged >5 years. Modified from: *Indinnimeo L* et al. *Gestione dell’attacco acuto di asma in età pediatrica Linea Guida SIP- Aggiornamento 2016*

As shown in the algorithm (Fig. 3), the treatment of an asthma attack depends on the severity of symptoms. There are no criteria on which to predict the evolution of an asthma attack, and the decision to hospitalize a child with an asthma attack should be taken based on the child’s clinical history, symptoms and lung function.

### Summary

**Table:** 

ᅟ	ᅟ
• Asthma is one of the most common causes of emergency department visits. • Treatment of asthma depends on the severity of the attack.	
• The severity of the asthma attack must be rapidly evaluated upon arrival in the emergency department.	
• Short-acting beta2-agonists represent the rescue medication of choice and should be taken as needed to reverse bronchoconstriction and relieve symptoms. • Oral corticosteroids should always be used in case of moderate and severe asthma attacks since they result in fewer and shorter hospitalizations. • According to international guidelines, a liquid formulation of oral corticosteroids is preferred to tablets in children.	

## Laryngotracheitis in children

Laryngotracheitis, also known as ‘viral croup’, is the most common and typical form of croup and refers to viral infection of the glottis and subglottic regions [68]. However, ‘croup’ is a generic term encompassing a heterogeneous group of childhood respiratory illnesses affecting the larynx, trachea and bronchi, characterized by barky cough, stridor, hoarseness and respiratory distress. These symptoms result from swelling in the area of the windpipe (trachea) just below the voice box (larynx). Laryngotracheal airway inflammation induces the typical symptoms in children because a small decrease in diameter secondary to mucosal edema and inflammation exponentially increases airway resistance and the work of breathing. During inspiration, the walls of the subglottic space are drawn together thereby producing the stridor characteristic of croup. Laryngotracheitis, laryngotracheobronchitis and spasmodic croup are included in the croup syndrome [69].

### Etiology and epidemiology

The most common causes of laryngotracheitis croup are parainfluenza viruses (types 1, 2, 3 and 4) and RSV. Other causative viruses are influenza A and B viruses, human metapneumovirus and adenovirus [70]. Laryngotracheitis is one of the most frequent causes of acute respiratory distress in young children. The disease is more common in boys than in girls and mainly affects children aged between 6 months and 3 years, with a peak annual incidence of almost 5% in the second year of life. However, it can occur in babies as young as 3 months old and in adolescents [71].

### Clinical history

Laryngotracheitis is often mild and self-limiting and resolves without any active intervention. However, there can be significant progressive inflammation and subglotting swelling that may lead to life-threatening airway obstruction. Symptom onset is typically abrupt and usually occurs at night. The child with croup presents a harsh cough, described as ‘barking’ or ‘brassy’, inspiratory stridor, hoarseness, low grade fever and respiratory distress that may develop slowly or quickly. Stridor is defined as the variably pitched noise of breathing associated with a partially obstructed upper airway. Inspiratory stridor occurs primarily with obstruction of the glottis but also with subglottic edema. These symptoms are frequently preceded by non-specific upper respiratory tract symptoms 12–48 h before development of the barky cough and difficulty breathing. Symptoms are generally short-lived, with resolution of the barky cough within 48 h in about 60% of children. These symptoms are very often worse at night, which may be a result of circadian fluctuations in endogenous serum cortisol, the concentrations of which peak at about 8 am and reach a trough between 11 pm and 4 am [71].

### Diagnosis

In the child with classic signs and symptoms, the diagnosis of croup is straightforward and can be made based on the history and physical examination alone. Routine laboratory tests do not help to establish the diagnosis. Ancillary testing should be reserved for the rare atypical presentations. Radiographic studies are rarely indicated and should be considered in a child in whom the diagnosis is unclear or who does not respond as expected to treatment. Anteroposterior radiographs of the neck can show the diagnostic subglottic narrowing of croup known as the ‘steeple sign’. However, radiographs should be considered only after airway stabilization (Table 6). According to the guideline developed by the Toward Optimized Practice Program, viral cultures and rapid antigen tests are not needed to confirm diagnosis [72].

**Table 6:** Radiological differential diagnosis of croup

Anteroposterior anterior neck radiograph can help to establish an alternative diagnosis in patients with atypical disease.	
• Cone-shaped narrowing instead of the normal squared shoulder appearance of the subglottic area suggests *croup.*	
• A ragged edge or a membrane spanning the trachea suggests *bacterial tracheitis.*	
• Thickening of epiglottis and aryepiglottic folds suggest *epiglottitis.*	
• Bulging of the posterior pharynx soft tissue suggests *retropharyngeal abscess.*	

* Reproduced from: Toward Optimized Practice (TOP) Working Group for Croup: Guideline for the diagnosis and management of croup. Alberta, Canada: Edmonton (AB); 2003 (revised 2008). [72]

### Differential diagnosis

The physician must be aware of other conditions that may present in a fashion similar to croup, namely, with symptoms of stridor and respiratory distress (Table 7). Generally, it helps to distinguish between infectious (epiglottitis, bacterial tracheitis and parapharyngeal abscess) and non-infectious causes of stridor (foreign body aspiration, allergic reaction, laryngomalacia, subglottic stenosis, hemangioma, vascular ring and vocal cord paralysis). It is particularly important to differentiate a presentation of croup from acute epiglottitis, which is a medical emergency due to the risk of sudden airway obstruction.

**Table 7:** Main clinical characteristics of laryngotracheitis, epiglottitis, bacterial tracheitis and spasmodic croup

Feature	Laryngotracheitis	Epiglottitis	Bacterial tracheitis	Spasmodic croup
Viral prodromal illness	++	-	+	+
Mean age	6–36 months	3-4 years	4-5 years	6–36 months
Illness onset	Gradual	Acute	Acute	Sudden
Fever	+/-	+	+	-
Quality of stridor	Harsh	Mild	Harsh	Harsh
Drooling, neck hyperextension	-	++	+	-
Cough	++	-	++	++
Sore throat	+/-	++	+/-	-
Recurrence	+	-	-	++
Hospitalization and intubation	Rare	Frequent	Frequent	Rare

From: Bell L: Middle respiratory tract infections. In: Pediatric Infectious Diseases: Principle and Practice. Edited by Jenson H, Baltimore R, 2nd edn. Philadelphia: Saunders; 2002: 772. [98]

Traditionally, researchers emphasized differences between spasmodic croup and laryngotracheitis (viral croup). Spasmodic croup describes a sudden onset of croup symptoms, usually at night, but without a significant upper respiratory tract prodrome. These episodes may be recurrent but are usually of short duration. It has been argued that spasmodic croup might be due to an allergic reaction to viral antigens rather than to a direct effect of viral infection [72].

### Management and treatment

**Fig. 4:**
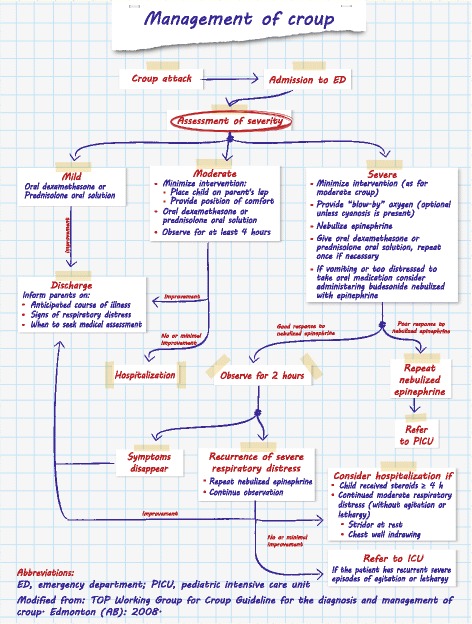
Management of croup. Modified from: *TOP Working Group for Croup Guideline for the diagnosis and management of croup. Edmonton (AB): 2008*

The most important assessment is the initial evaluation of croup severity, which is based on assessment of respiratory status and rate, chest wall retractions, stridor, heart rate, use of accessory muscles and mental status (Fig. 4). The Westley croup score (WCS) is the most widely used system with which to evaluate the severity of this disorder (Table 8). The extent of airway obstruction is also classified as mild, moderate or severe.

**Table 8:** Westley croup score

Stridor	None: 0
	With agitation: +1
	At rest: +2
Chest wall retractions	None: 0
	Mild: +1
	Moderate: +2
	Severe: +3
Cyanosis	None: 0
	With agitation: +4
	At rest: +5
Level of consciousness	Normal: 0
	Disoriented: +5
Air entry	Normal: 0
	Decreased: +1
	Markedly decreased: +2

*Mild croup* (WCS ≤2): occasionally barky cough, no audible stridor at rest, and no to mild suprasternal and/or intercostal indrawing (retractions of the skin of the chest wall)

*Moderate croup* (WCS 3-5): frequent barky cough, easily audible stridor at rest, and suprasternal and sternal wall retractions at rest, but no or little distress or agitation

*Severe croup* (WCS 6-11): frequent barky cough, prominent inspiratory and occasionally expiratory stridor, marked sternal and wall retractions, and significant distress and agitation

*Impending respiratory failure* (WCS >11): barky cough, audible stridor at rest, sternal and wall retractions, lethargy or decreased level of consciousness and often dusky appearance without supplemental oxygen

In terms of general care, there is a consensus that children with croup should be made as comfortable as possible, and clinicians should take particular care not to frighten children during treatment because agitation may greatly worsen symptoms. The best way to lessen agitation is to examine and treat the child while he/she is sitting comfortably in the lap of a parent. Oxygen should be administered if the child is in respiratory distress (for the management of children requiring ventilation, see below). Although used for decades in the acute care setting, humidified air (mist) is now recognized to be ineffective in croup and should not be given [73]. Heliox was found to improve clinical croup scores in some small trials, but there is no evidence of significant clinical improvements versus standard treatments [74]. Similarly, antibiotics, oral decongestants and sedation are not indicated.

Conventionally, croup is treated with corticosteroids and epinephrine. The algorithm (Fig. 4) summarizes the acute management of croup. Corticosteroid therapy benefits patients with croup by decreasing edema in the laryngeal mucosa, and steroids play a part in the management of croup regardless of severity.

Dexamethasone and prednisolone are the most commonly used glucocorticoids and are the most effective for mild-to-moderate croup. Oral corticosteroids are well absorbed and reach peak serum concentrations as rapidly as corticosteroids administered intramuscularly (and without pain). Indeed, oral and intramuscular administration yield equivalent results [75]. There is no evidence that multiple doses provide additional benefit over a single dose. Nebulized budesonide is not routinely indicated for the treatment of croup except in the cases of a child with severe respiratory distress and of a child who has had persistent vomiting. In these patients, budesonide may be mixed with epinephrine and administered simultaneously.

Nebulized epinephrine causes vasoconstriction of the subglottic mucosa which reduces edema and swelling. In a systematic review, the routine use of nebulized epinephrine was found to provide rapid, short-term relief of severe respiratory distress [76]. The recommended dose is 0.05 mL/kg (maximal dose 0.5 mL) of racemic epinephrine 2.25%, or 0.5 mL/kg (maximal dose 5 mL) of L-epinephrine 1:1,000 via nebulizer in the clinical setting. Racemic epinephrine has traditionally been used to treat children with croup. However, L-epinephrine 1:1,000 is as effective and safe as the racemate form [76]. The clinical effects of nebulized epinephrine are sustained for at least 1 h. It is important to underline that the patient’s symptoms return as the effect of epinephrine wears off. The administration of nebulized epinephrine, one dose at a time, in children has not been associated with such adverse effects as a significant increase in heart rate or blood pressure [76].

In conclusion, oral corticosteroids are the treatment of choice for children with croup whereas nebulized epinephrine is indicated for children with severe respiratory distress. This therapy substantially decreases intubations, hospital admissions and return visits for medical care.

### Summary

**Table:** 

ᅟ	ᅟ
• Laryngotracheitis, also known as ‘viral croup’, is the most common and typical form of croup, and refers to viral infection of the glottis and subglottic regions. • The child with laryngotracheitis presents a harsh cough, described as ‘barking’ or ‘brassy’, inspiratory stridor, hoarseness, low grade fever and respiratory distress that may develop slowly or quickly. • Severity can be assessed by the Westley croup score (WCS). • Anteroposterior radiographs of the neck are rarely indicated and should be considered in a child in whom the diagnosis is unclear or who does not respond as expected to treatment. • Laryngotracheitis is often mild and self-limiting and resolves without any active intervention. • Oral corticosteroids are the treatment of choice for children with mild-to-moderate croup whereas inhaled corticosteroids and nebulized epinephrine are indicated for children with severe respiratory distress.	

## Treatment of acute respiratory failure in children

Acute lower respiratory tract disease (LRTD) is a leading cause of morbidity and mortality in children < 5 years of age and, notably, children are more susceptible than adults to severe manifestations of respiratory diseases, which in some cases lead to blood oxygen desaturation. The higher susceptibility of infants may be explained by differences in respiratory physiology between children and adults (see Table 9).

**Table 9:** Differences in respiratory physiology between children and adults

Cause	Physiological or anatomical basis
Metabolism ↑	O_2_ consumption ↑
Risk of apnea ↓	Immaturity of control breathing
*Airway resistance ↑*	
Upper airway resistance ↑	Nose breathing
	Large tongue
	Airway size ↓
	Collapsibility ↑
	Pharyngeal muscle tone ↓
	Compliance of upper airway structure ↑
Lower airway resistance ↑	Airway size ↓
	Collapsibility ↑
	Airway wall compliance ↑
	Elastic recoil ↓
Lung volume ↓	Numbers of alveoli ↓
	Lack of collateral ventilation
Efficiency of respiratory muscles ↓	Efficiency of diaphragm ↓
	Rib cage compliance ↑
	Horizontal insertion at the rib cage
	Efficiency of intercostal muscles ↓
	Horizontal ribs
Endurance of respiratory muscles ↓	Respiratory rate ↑
	Fatigue-resistant type I muscle fibres ↓

From: HammerJ, Eber E (Eds) Paediatric pulmonary function testing. Prog Respir Res. Basel, Karger, Vol 33, 2005. [99]

Hypoxemia, defined as a decrease in the partial pressure of oxygen in the blood, can be caused in LRTD by hypoventilation, ventilation-perfusion mismatch, right-to-left shunt, diffusion impairment, or reduced inspired oxygen tension [77]. Hypoxemia associated with more severe acute LRTDs is a major criterion for hospitalization and is more common in young patients.

Respiratory failure is divided into two types:

- Type 1, which is defined as hypoxemia without hypercapnia. Conditions more often leading to type 1 respiratory failure are characterized by altered ventilation/perfusion.
- Type 2, which is determined by hypoxia with hypercapnia. The most frequent causes are increased airway flow resistance and a decreased surface for gas exchange.

### Management

#### Hypoxemia and normocapnia

When hypoxemia is the only complication of severe LRTD and no hypercapnia is identified, administration of simple oxygen is indicated to maintain blood oxygen levels. Although, multiple clinical practice guidelines and protocols recognize that oxygen therapy is an important component of the treatment of severe acute LRTD [78], there are no specific guidelines on the correct method of administering oxygen. Many non-invasive oxygen-delivery appliances are available; the most frequently used devices are:

- *Face mask*. This is connected to an oxygen source and placed over the patient’s nose and mouth. At high oxygen flow rates, room air can be entrained through the small perforations in the mask, whereas low-flow rates may lead to carbon dioxide retention. The concentration of oxygen delivered varies depending on two factors: the patient’s respiratory flow rate and oxygen flow [79]. A face mask interferes with feeding.
- *Hood*. This is a transparent plastic box placed around the infant’s head [80]. It requires high oxygen flow rates (>5 L/min) to prevent re-breathing of carbon dioxide [79]. This method enables delivery of a specific fraction of inspired oxygen (FiO_2_). It limits the infant’s mobility and interferes with feeding.
- *Nasal cannulae or prongs.* These devices deliver oxygen directly into the patient’s nostrils. They can be either low-flow or high-flow (HFNC) and humidification is required with a flow rate >4 L/min [81]. In low-flow oxygen treatment, FiO_2_ varies in relation to the patient and to the type of prongs, and it is not possible to determine the amount of FiO_2_ reaching the patient’s airway [79, 80]. On the contrary, with HFNC, the exact amount of FiO_2_ reaching the airway can be calculated and modulated irrespective of oxygen flow.

Non-hypercapnic hypoxemia in children with LRTD has been associated with an increased risk of mortality and long-term morbidity [82]. Consequently, it is feasible that supplemental oxygen therapy may improve the outcomes of hypoxemic children presenting with LRTD.

More severely ill patients with significant respiratory distress, high work of breathing and an enhanced risk of hypercapnia may benefit from other respiratory methods of support, namely, HFNC, helmet ventilation or continuous positive airway pressure (CPAP), which share the same pathophysiological principle. Continuous slightly heated humidified air pressure maintains the airways open and prevents alveolar collapsing thereby recruiting more of the lung's surface area for ventilation. Moreover, heated humidification of the respiratory gas facilitates secretion clearance. Thanks to these mechanisms, HFNC, helmet ventilation and CPAP improve the patient’s oxygenation, gas exchange and reduce the work of breathing, which is important in young children affected by acute respiratory failure.

*HFNC* consists in the administration of a heated and humidified mixture of air and oxygen at a flow rate higher than the patient’s inspiratory flow. Although ‘high-flow’ has yet to be defined, flow rates >2 L/min are generally considered high in infants, and flow rates >6 L/min are considered high in children [83]. In clinical practice, some authors suggest adjusting the oxygen flow rates according to body weight, and recommend a flow rate of 1 L/kg/min (max 2 L/kg/min), which provides a degree of distending pressure [84, 85] and reduces the work of breathing [86]. Oxygen fraction via blender must be frequently adjusted to maintain target oxygen saturation ≥92. FiO2 can be gradually weaned when the clinical condition improves as witnessed by decreased work of breathing and improved respiratory rate. There is no need to wean flow rate (although this may be considered if flow rates above 1 L/kg/min have been instituted) [87].

*The helmet device*, particularly in younger children, ensures slightly higher pressure values (around 5 cmH_2_O) compared to HFNC. The helmet, placed around the patient’s head, must be attached to the patient by a specific fixing system. Helmets are equipped with an automatic anti-choking system, and a porthole that gives airtight access to the patient [88].

CPAP ventilation (pressures vary between 5 and 12 cm H2O) does not actively assist inspiration; in fact the patient must sustain the work of breathing. Thus, CPAP ventilation cannot be considered a true ventilation mode [89–91]. Continuous positive pressure keeps the airways open, promotes relaxing of the upper airway dilator muscles, and reduces the activity of the inspiratory muscles of the upper airways and diaphragm [89, 91].

It is important to stress that the above-described methods of acute respiratory support should be started only:

- In a pediatric setting in which the patient’s clinical course can be closely monitored.
- If there is a sufficient number of staff well trained to recognize the early signs of respiratory failure.

#### Hypoxemia and hypercapnia

Children with hypercapnic respiratory failure associated with a poor oxygen-carbon dioxide exchange must be treated with ventilation. Although it is reasonable to attempt non-invasive ventilation (NIV), young patients with severe acute respiratory failure that require respiratory support might need invasive, positive pressure mechanical ventilation, either conventional or high-frequency. Invasive ventilation bypasses the patient’s upper airway with an artificial airway (i.e., an endotracheal tube, a laryngeal mask or tracheostomy tube). Multiple mechanical ventilation modes are currently used in clinical practice to provide respiratory support for a wide spectrum of patients.

As mentioned above, whenever possible and safe, NIV is the method of choice, as it doesn’t disrupt swallowing, feeding, speaking or coughing. In pediatric practice, NIV has reduced the number of children needing intubation and has also helped to reduce the length of stay in pediatric intensive care units thanks to the shorter weaning time versus invasive ventilation [92]. Unlike invasive ventilation, NIV preserves the vocal cords and trachea, and reduces the risk of infection [93].

The major short-term goals for children with type 2 acute respiratory failure treated with NIV are to obtain immediate relief from symptoms, reduce the work of breathing, improve and stabilize gas exchanges, optimize the level of comfort and avoid intubation [94]. Like oxygen treatment, NIV can be practiced with various interfaces. The medical choice depends on the characteristics of the patient (age, facial characteristics, degree of cooperation and severity of respiratory impairment). Physicians should bear in mind that the effectiveness of ventilation is inversely correlated to air leaks [90]. In children, acceptance of the interface is the first step to successful NIV [95]. Interfaces should have good adhesion, a low resistance to airflow, be light, exert the lowest pressure on the skin compatible with effective ventilation, and the dead space volume should be minimized [96]. Nasal masks are still the most widely used interfaces in children, but oro-nasal and full-face masks are increasingly being used in many specialized centers [96, 97]. A see-through mask is a good option as it enables the physician to verify correct positioning of the mask and to identify complications such as sudden vomiting. Nasal pillows and mouthpieces are other possible options.

As already mentioned, frequent monitoring during NIV is necessary to ensure the effectiveness and safety of the procedure. The level and type of monitoring should be proportional to the patient’s clinical condition [90, 91]. Patients being treated acutely should be continuously monitored in hospital with a pulse-oximeter or a multichannel cardio-respiratory monitor. Close clinical observation is also mandatory and must include assessment of respiratory rate and fatigue, level of dyspnea, signs of patient-ventilator asynchrony, air leaks or short-term complications of NIV. Side effects of NIV in acute treatment, such as cutaneous lesions, gastric distension and dry eyes, are uncommon and described as ‘minor’. Arterial blood gas analysis should be assessed 1–4 h after the onset of NIV and 1 h after each change in the ventilator setting or FiO2 concentration [90, 91].

### Summary

**Table:** 

ᅟ	ᅟ
• Because of differences in respiratory physiology, children are more susceptible than adults to severe manifestations of respiratory diseases, which in some cases lead to blood oxygen desaturation. • Hypoxemia associated with severe respiratory diseases is a major criterion for hospitalization and is more common in young patients. • Administration of simple oxygen is indicated when hypoxemia is the only complication and no hypercapnia is present. • Patients with significant respiratory distress may benefit from HFNC, helmet ventilation or CPAP. These procedures must be closely monitored and performed by trained staff. • If ventilatory support is needed, NIV is the method of choice whenever possible and safe. It doesn’t disrupt swallowing, feeding, speaking or coughing, and preserves the vocal cords and trachea. It also reduces the risk of infection and the length of stay in pediatric intensive care units.	

## Conclusions

Thanks to their anti-inflammatory action, corticosteroids are widely used to treat respiratory diseases in pediatric practice. However, the decision whether or not to administer a glucocorticoid in case of acute respiratory disease must be made based on evidence of efficacy and according to guidelines. There are no convincing data supporting the use of corticosteroids to treat bronchiolitis. Oral corticosteroids may be beneficial in preschool children with severe wheezing exacerbations that require emergency department or hospital admission, but currently they are not indicated in preschool children affected by mild exacerbation of viral wheeze. In case of moderate and severe asthma attacks, all clinical guidelines agree that oral corticosteroids should be administered since they result in fewer and shorter hospitalizations. In particular, guidelines recommend oral prednisolone for the early treatment of acute asthma exacerbations. Finally, oral corticosteroids are the treatment of choice for children with mild-to-moderate croup.

Although corticosteroids exert a beneficial effect on the course of different acute respiratory diseases in childhood, the type of corticosteroid to administer and the appropriate dosage should be carefully evaluated in order to minimize potential adverse effects.

## Abbreviations

ACTH: Adrenocorticotropin
CPAP: Continuous positive airway pressure
GINA: Global Initiative for Asthma
HFNC: High-flow nasal cannula
HPA: Hypothalamic-pituitary-adrenal
HRV: Human rhinovirus
LRTD: Lower respiratory tract disease
NIV: Non-invasive ventilation
RSV: Respiratory syncytial virus
SABAs: Short-acting beta2-agonists
SC: Systemic corticosteroids
SIAIP: Italian Society of Pediatric Allergology and Immunology
SIDRIA: Italian Studies on Respiratory Disorders in Childhood and the Environment
SIMRI: Italian Society of Pediatric Respiratory Diseases
TOP: Toward optimized practice
WCS: Westley croup score
